# Supplementary material for: Identification of Novel Senescent Markers in Small Extracellular Vesicles
Source: Int J Mol Sci. 2023 Jan 26;24(3):2421. doi: 10.3390/ijms24032421 (PMC9916821; doi:10.3390/ijms24032421)
Supplement: Supplementary file 1 [file ijms-24-02421-s001.zip › Supplementary Materials/Supplementary Figures S1-S9.pdf]

Supplementary Materials for

Identification of novel senescent markers in small extracellular vesicles

Tomoka Misawa, Kazuhiro Hitomi, Kenichi Miyata, Yoko Tanaka, Risa Fujii, Masatomo Chiba, Tze Mun Loo, Aki Hanyu, Hiroko Kawasaki, Hisaya Kato, Yoshiro Maezawa, Koutaro Yokote, Asako J. Nakamura, Koji Ueda, Nobuo Yaegashi, Akiko Takahashi*

This supplementary file includes:

Figure S1 to S9

Legends for Table S1 to S3

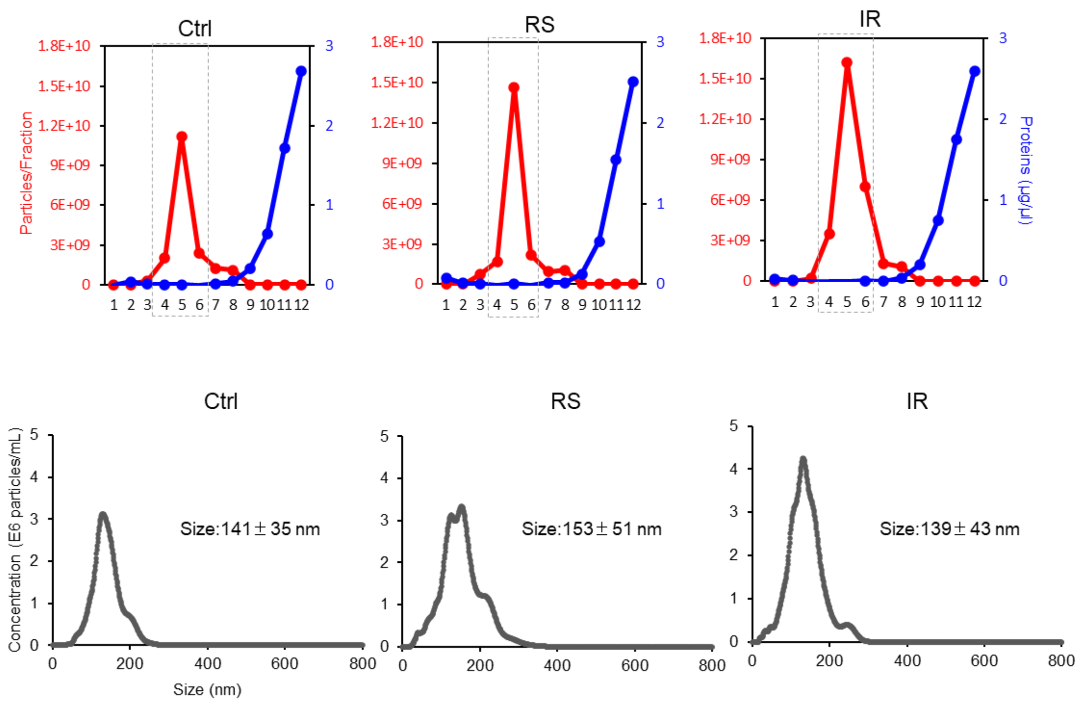

Figure S1.

Nanoparticles tracking analysis (NTA) and protein quantitation assay for quantitative measurement of sEVs collected by size exclusion chromatography (SEC). The horizontal axis shows each fraction number. The left vertical axis is the number of particles for each fraction and the right vertical axis is the protein concentration (upper panel). Particle size distribution analyzed by NTA (lower panel).

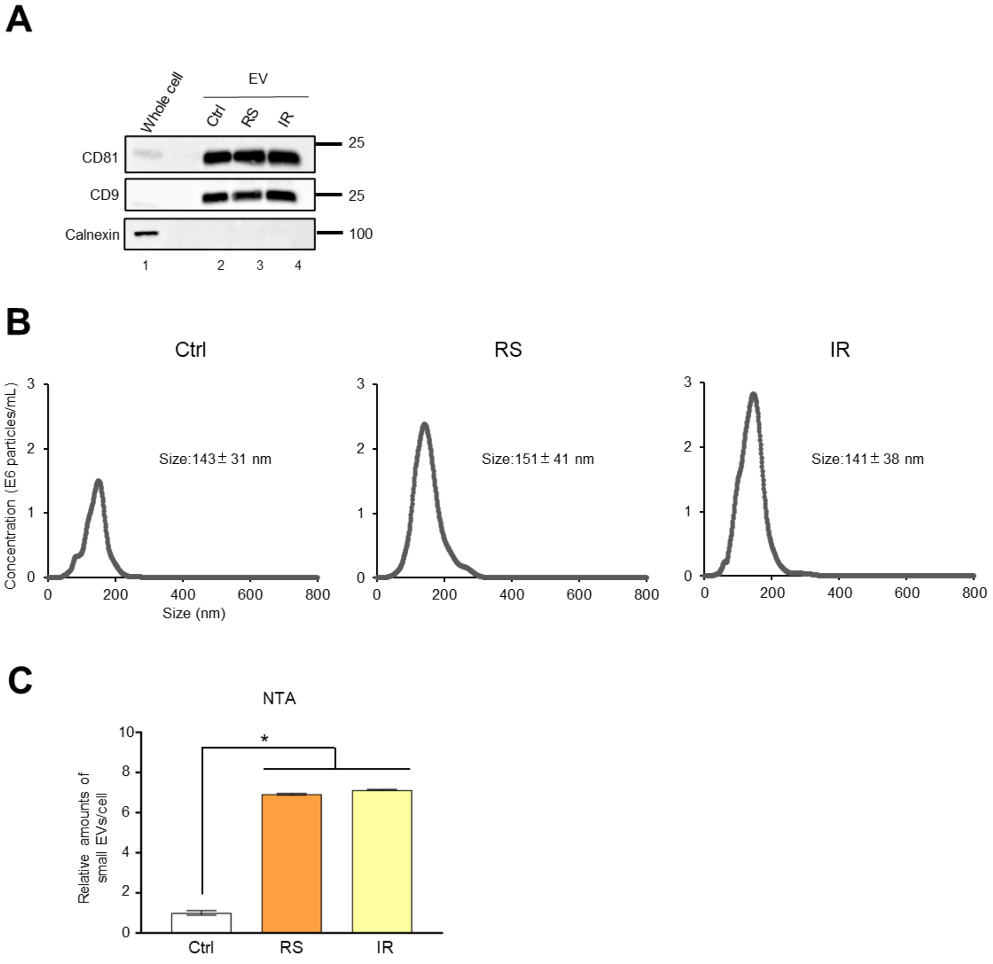

Figure S2.

(A) Western blot analysis of exosome markers CD9, CD81 and exosome negative marker calnexin. **(B)** NTA for quantitative measurement of sEVs collected by phosphatidylserine affinity columns. **(C)** The histograms indicate the relative amounts of sEVs per cell. Statistical analysis was performed using a two-tailed unpaired Student's t-test (*$P < 0.05$ by the unpaired two-sided t-test).

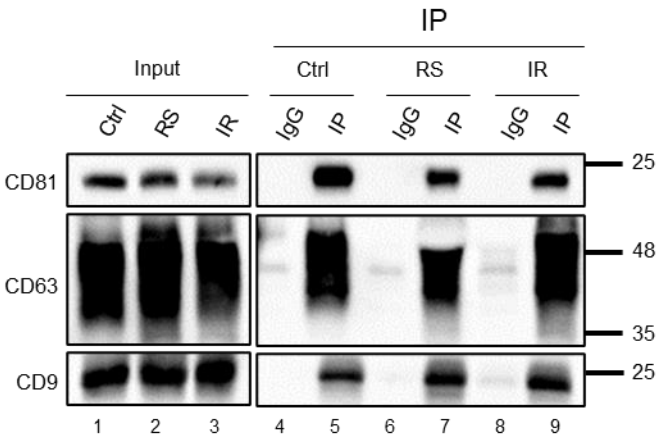

Figure S3.

Western blot analysis of exosome markers CD81, CD63, and CD9 for quantitative measurement of sEVs collected by IP.

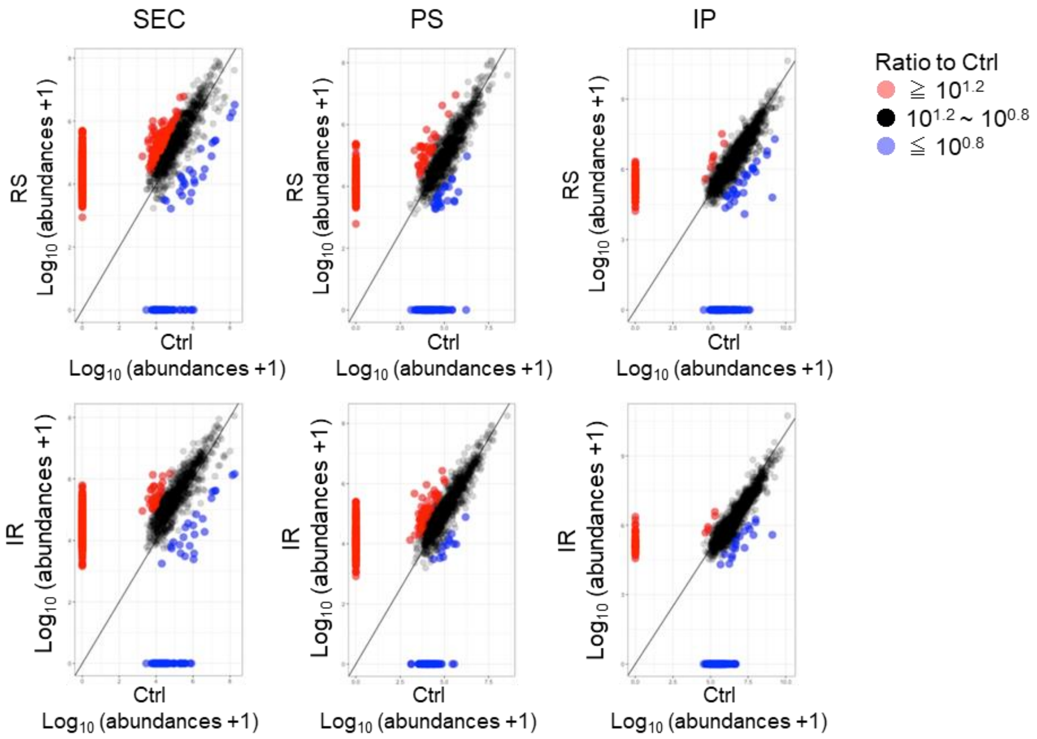

Figure S4.

Scatter plots showing the abundances calculated by mass spectrometry of sEVs isolated from conditioned medium derived from control (Ctrl) and RS (upper panels), or Ctrl and IR (lower panels) TIG-3 cells using the three isolation methods. The red dots represent proteins that are $10^{1.2}$ times higher than the Ctrl, the blue dots represent proteins that are $10^{0.8}$ times lower than the Ctrl.

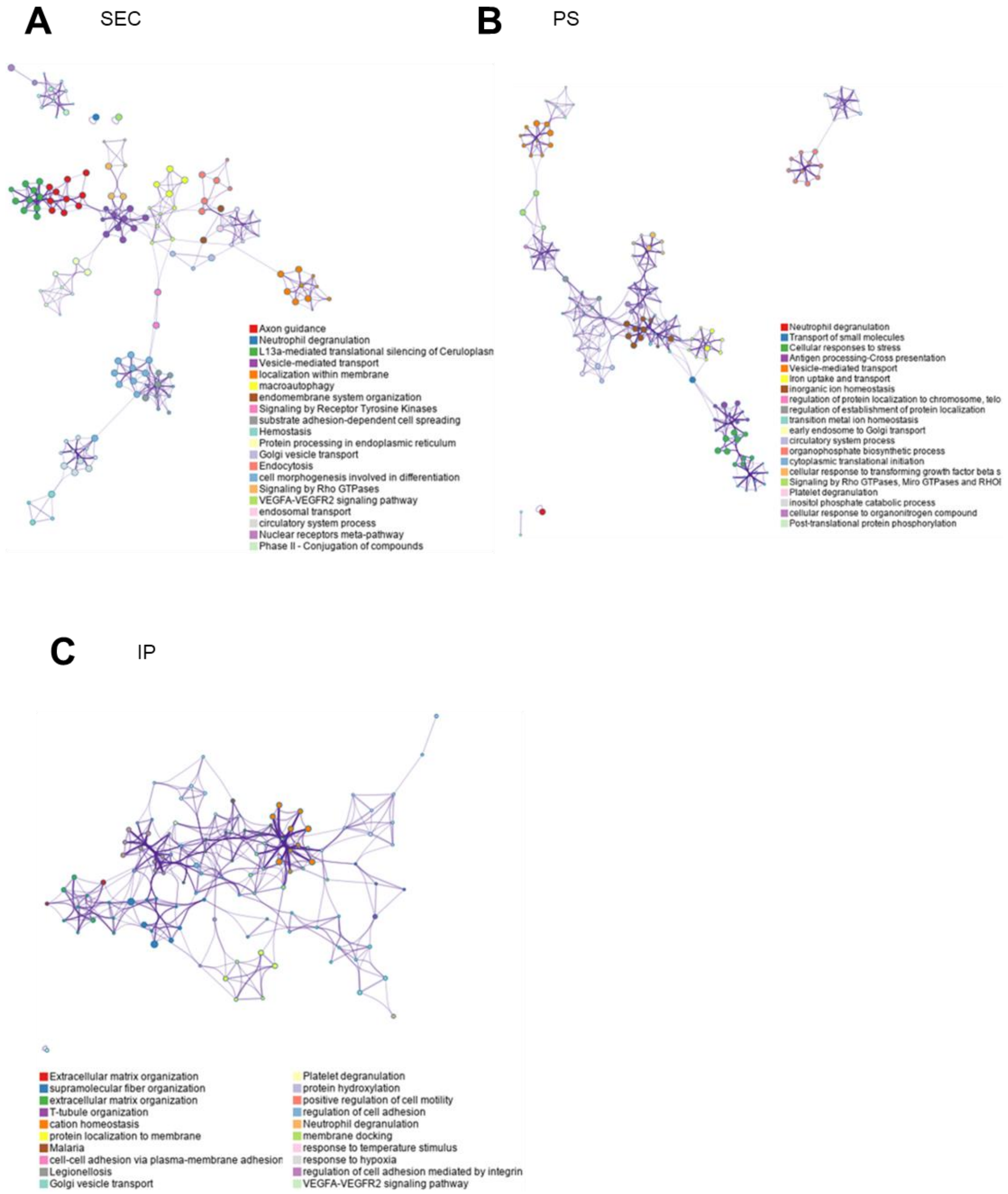

Figure S5.

(A-C) Pathway and Process Enrichment Analysis performed by Metascape on proteins detected specifically in sEVs isolated from CM derived from senescent TIG-3 cells collected by the three methods. **(A)** Size exclusion chromatography (SEC), **(B)** Affinity column for phosphatidylserine (PS), and **(C)** Immunoprecipitation (IP) using antibodies against tetraspanin proteins (CD9, CD63, and CD81).

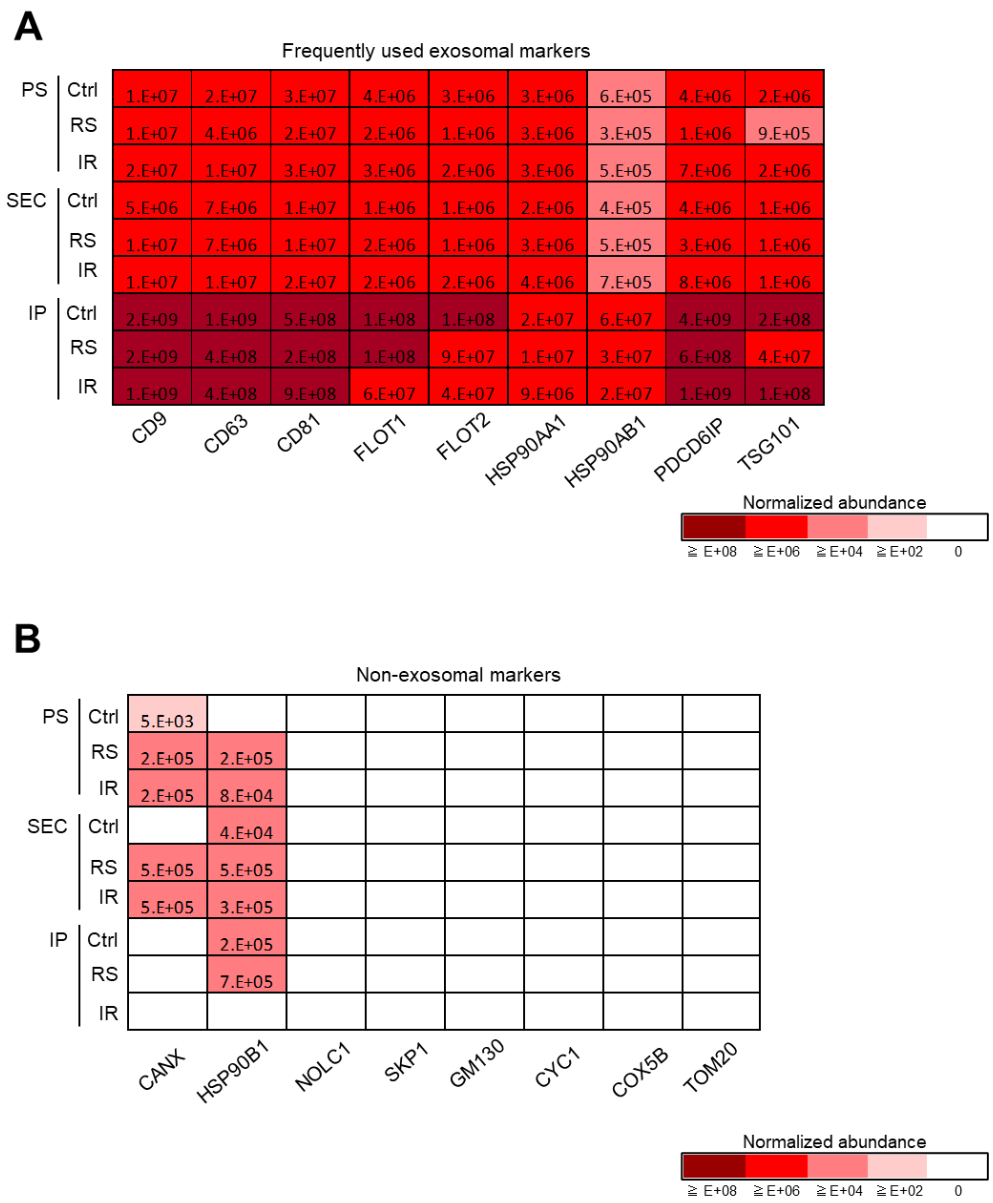

Figure S6.

(A) The normalized abundance of frequently used exosomal marker proteins in the sEVs collected by the three methods. **(B)** The normalized abundance of the frequently used exclusion marker calnexin and the non-exosomal markers. Proteins not detected by MS analysis are shown as a white box.

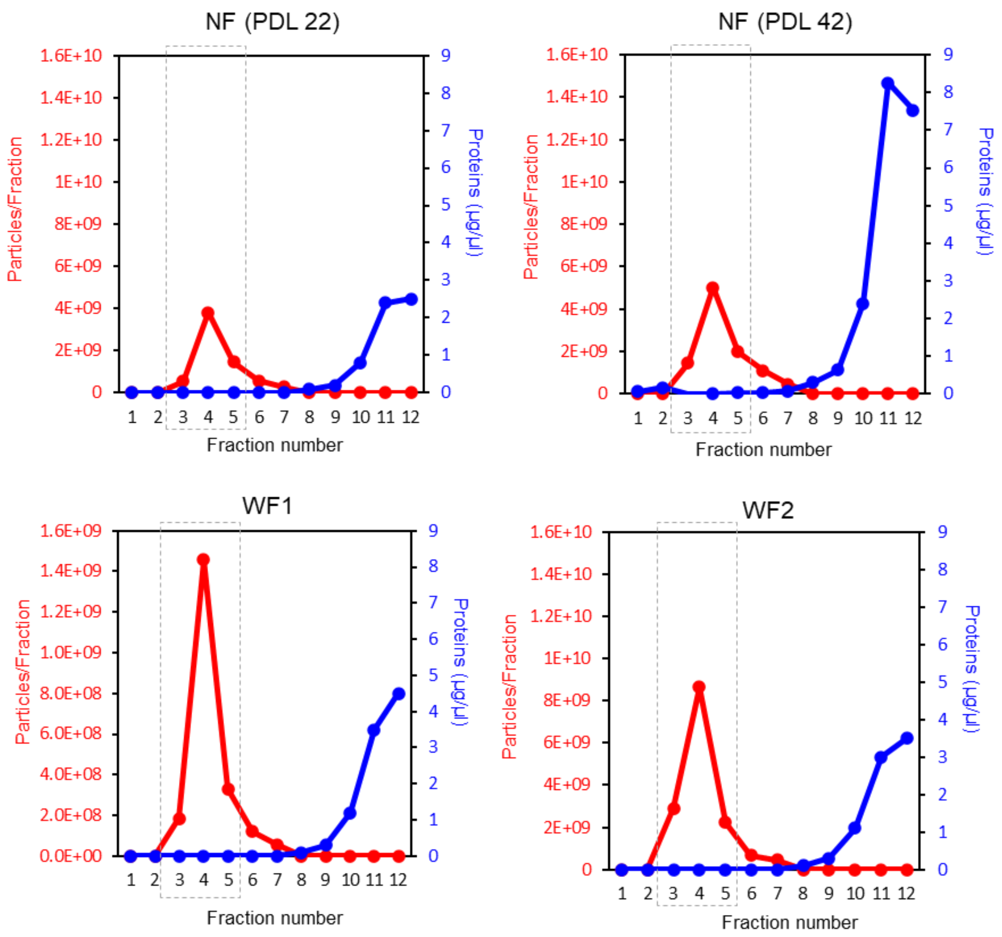

Figure S7.

Nanoparticles tracking analysis (NTA) and protein quantitation assay for quantitative measurement of sEVs collected by SEC from the fibroblasts of Werner syndrome patients and age-matched volunteers. The horizontal axis shows each fraction number. The left vertical axis is the number of particles for each fraction and the right vertical axis is the protein concentration.

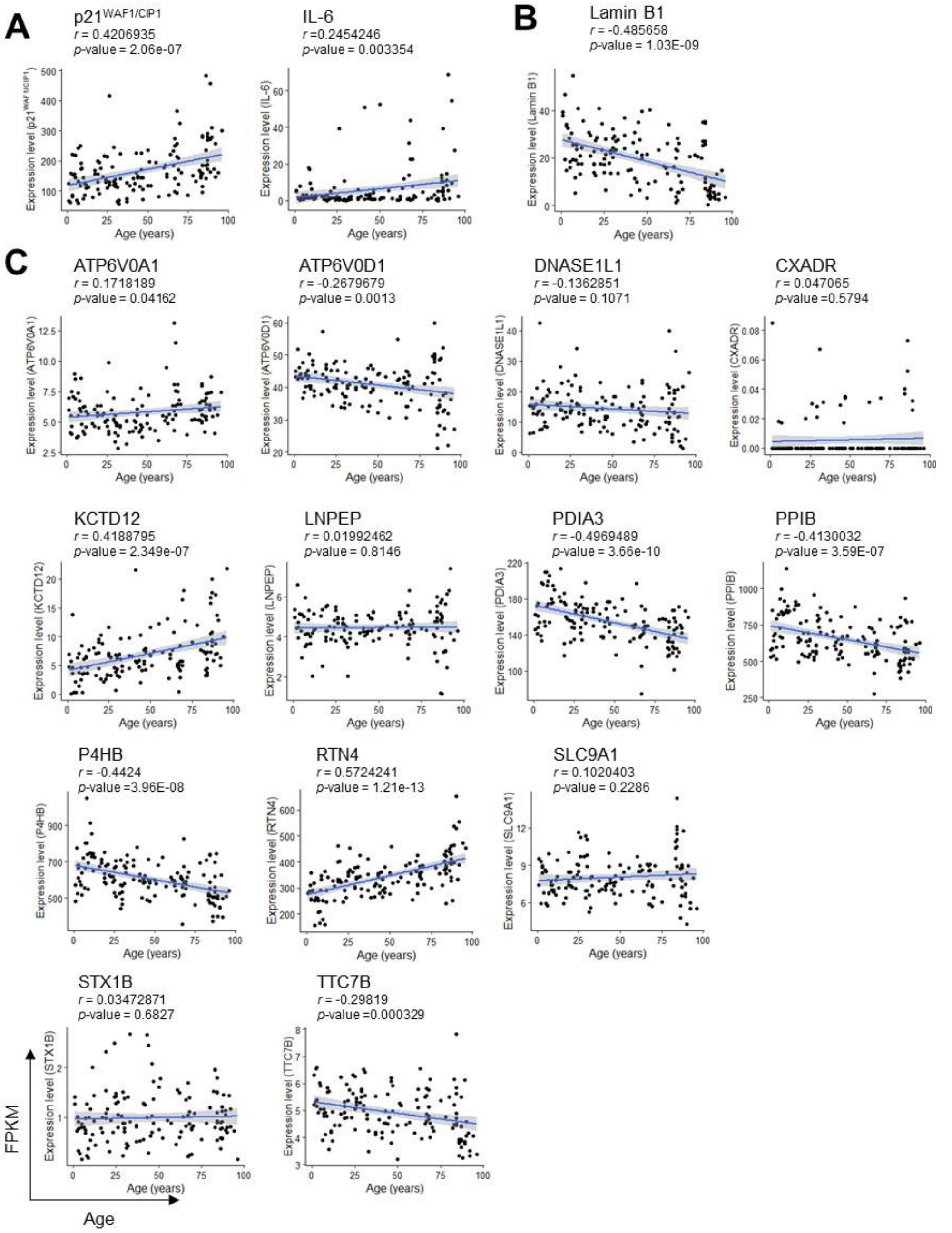

Figure S8.

(A-C) The correlation between gene expression levels of senescence markers (p21WAF1/CIP1 and IL-6) **(A)**, lamin B1 **(B)**, and thirteen genes **(C)** and age using the previous RNA sequence data from human dermal fibroblasts collected from 1 to 94 year-old subjects (GSE113957, [39]). *P*-value < 0.01

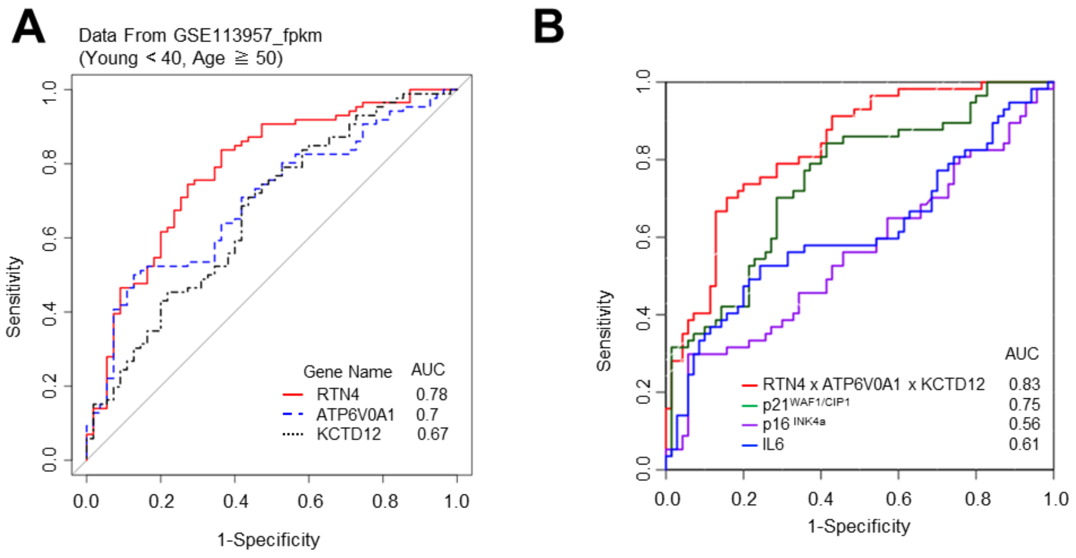

Figure S9.

ROC curves for identification of the sensitivity, specificity, and the area under the curve (AUC) of marker expression in young (n = 65) vs. aged (n = 62) human dermal fibroblasts. **(A)** The receiver operating characteristic (ROC) curves of the three proteins (ATP6V0A1, KCTD12, or RTN4) to that distinguish young individuals (< 40 years old) from that of aged individuals (≧ 50 years old). AUC: area under the curve. **(B)** The combination biomarker model, ATP6V0A1×KCTD12×RTN4, was also evaluated by logistic regression analysis and compared with the predictive ability of major senescence markers ($p21^{WAF1/CIP1}$, $p16^{INK4a}$, and IL-6).

Supplementary Table Legends

Table S1. Process and enrichment analysis data, related to Supplementary Figure 5. A-C columns are the results of pathway analysis of proteins specifically enriched ($P < 0.01$) in senescent cells (RS/IR) by sEVs purified by SEC. E-G columns are the results of Pathway analysis of proteins specifically Enrich ($P < 0.01$) in senescent cells (RS/IR) with sEVs purified by PS. I-K columns are the results of Pathway analysis of proteins specifically Enrich ($P < 0.01$) in senescent cells (RS/IR) with sEVs purified by IP. R-S columns are the results of pathway analysis of proteins commonly enriched ($P < 0.01$) in senescent cells (RS/IR) specifically in the three conditions.

Table S2. Related to Figure 2 and Figure 3. The list of abundance ratio of senescent EVs to Ctrl-EVs, which collected by the three methods, and the list of abundance ratio of WF cell-derived EVs to NF cell-derived EVs, which collected by SEC method. Proteins with unique peptides > 2 and abundance ratio > 2 were extracted.

Table S3. Related to Figure 4E. The list of normalized abundance of thirteen proteins common to sEVs collected by the three methods in serum-derived EVs of mice. Bolded proteins are significantly more abundant in serum-derived EVs from aged mice than in serum-derived EVs from young mice.
